# Extravascular lung water index measurement in critically ill children does not correlate with a chest x-ray score of pulmonary edema

**DOI:** 10.1186/cc9054

**Published:** 2010-06-08

**Authors:** Joris Lemson, Lya E van Die, Anique EA Hemelaar, Johannes G van der Hoeven

**Affiliations:** 1 Department of Intensive Care Medicine, Radboud University Nijmegen Medical Centre, Nijmegen. PO box 9101, 6500 HB Nijmegen, The Netherlands; 2 Department of radiology, Radboud University Nijmegen Medical Centre, Nijmegen. PO box 9101, 6500 HB Nijmegen, The Netherlands

## Abstract

**Introduction:**

Extravascular lung water index (EVLWI) can be measured at the bedside using the transpulmonary thermodilution technique (TPTD). The goal of this study was to compare EVLWI values with a chest x-ray score of pulmonary edema and markers of oxygenation in critically ill children.

**Methods:**

This was a prospective observational study in a pediatric intensive care unit of a university hospital. We included 27 critically ill children with an indication for advanced invasive hemodynamic monitoring. No specific interventions for the purpose of the study were carried out. Measurements included EVLWI and other relevant hemodynamic variables. Blood gas analysis, ventilator parameters, chest x-ray and TPTD measurements were obtained within a three-hour time frame. Two radiologists assessed the chest x-ray and determined a score for pulmonary edema.

**Results:**

A total of 103 measurements from 24 patients were eligible for final analysis. Mean age was two years (range: two months to eight years). Median cardiac index was 4.00 (range: 1.65 to 10.85) l/min/m^2^. Median EVLWI was 16 (range: 6 to 31) ml/kg. The weighted kappa between the chest x-ray scores of the two radiologists was 0.53. There was no significant correlation between EVLWI or chest x-ray score and the number of ventilator days, severity of illness or markers of oxygenation. There was no correlation between EVLWI and the chest x-ray score. EVLWI was significantly correlated with age and length (r^2 ^of 0.47 and 0.67 respectively).

**Conclusions:**

The extravascular lung water index in critically ill children does not correlate with a chest x-ray score of pulmonary edema, nor with markers of oxygenation.

## Introduction

Extravascular lung water index (EVLWI) can be measured at the bedside using the transpulmonary thermodilution technique (TPTD) incorporated in the PiCCO device (Pulsion, Munich, Germany). Besides EVLWI, the TPTD technique also measures cardiac output (CO) and global end diastolic volume index (GEDVI). EVLWI reflects the amount of fluid present in the pulmonary interstitium and probably also in the alveolar space while GEDVI is a reflection of the blood volume of the heart and intrathoracic great vessels. Consequently, GEDVI is used as an index for cardiac preload [1,2].

In adults, EVLWI measurement using the TPTD technology reflects pulmonary edema and correlates with severity of illness or outcome [3-10]. An EVLWI between 3 and 7 ml/kg is considered normal in adults. Levels above 10 ml/kg are associated with clinical pulmonary edema [7]. EVLWI divided by GEDVI may distinguish between pulmonary edema due to increased capillary permeability or increased hydrostatic pressure [11,12]. Furthermore therapy driven by EVLWI measurements may improve outcome [13-15].

We previously showed that the TPTD technique is reliable in children when compared to the clinical gold standard, the double indicator dilution technique using injections of ice-cold indocyanine green [16]. However, measured EVLWI values are higher compared to adults, especially in younger children [16-18]. Since fluid overload is also related to poor outcome in children it could be advantageous to use the EVLWI measurement for quantification of (pulmonary) edema [19,20] and as a guide for directing therapy [13-15].

The presence and quantity of pulmonary edema in children are usually determined with the bedside chest x-ray. Also, oxygenation parameters like PaO_2_/FiO_2 _(P/F ratio) and A-a gradient reflect the severity of pulmonary edema and thus EVLWI. Up to date EVLWI measurements in critically ill children in relation to parameters of oxygenation have not been studied.

The goal of this study was to compare the EVLWI with a chest x-ray score of pulmonary edema in a general critically ill pediatric population. Furthermore, we compared both the EVLWI and the chest x-ray score with collected markers of oxygenation and severity of illness scores.

## Materials and methods

### Patients

We included 27 consecutive mechanically ventilated critically children <10 years admitted to our pediatric intensive care unit with an indication for advanced hemodynamic monitoring. Fluid loading or vasoactive support was used according to the judgment of the treating physician. Mechanical ventilation was performed using an oral or nasal, cuffed or uncuffed endotracheal tube with a Servo 300 ventilator (Maquet, Sweden). Patients were monitored with a 3 French 7 cm arterial Pulsiocath (Pulsion, Munich, Germay) catheter in the femoral position. Central venous access was accomplished using standard venous catheters in femoral, subclavian or jugular position without echo guidance. No extra catheters were inserted for study purposes only. PICU treatment was not influenced by the data obtained from this study. Because of the observational nature the local ethics committee responsible for medical research in humans approved the study and waived the need for informed consent.

### Data collection

We collected patient demographics, admission diagnosis, length of PICU stay, number of ventilation days and severity of illness scores (PIM and PRISM II). When a chest x-ray was ordered we measured EVLWI and other hemodynamic parameters using the TPTD technique. Arterial blood gas analysis was performed and ventilation parameters were collected all within a three-hour time frame. Measurements were not performed if a rapid change in blood pressure, cardiac output or heart rate occurred. Ventilator settings and the dose of vasoactive drugs were not changed during this period.

TPTD measurements were performed using the PiCCOplus or PiCCO_2 _device and included CO, EVLWI, GEDVI and the ratio of EVLWI to GEDV. Other recorded hemodynamic parameters were heart rate (HR), systolic, diastolic, mean invasive blood pressure (SAP, DAP and MAP) and central venous pressure (CVP). Ventilator data included the type of ventilation, inspiratory oxygen fraction (FiO_2_), positive end expiratory pressure (PEEP) level and peak pressure. Arterial blood gases were drawn in a standard way and sent to the laboratory for routine evaluation. We calculated the P/F ratio and the alveolar arterial oxygen gradient (A-a gradient) using standard formula and a respiratory quotient (RQ) of 0.8.

### TPTD measurements

The TPTD technology has been described in detail elsewhere [7,16,21,22]. The measurement of CO, EVLWI and GEDVI is based upon the properties of the transpulmonary thermodilution curve. The area under the dilution curve represents CO. The time interval between injection and passage of the indicator (Mean Transit time) represents intrathoracic blood volume and the rate of decline of the dilution curve (Down Slope time) the amount of extravascular lung water. The calculation of EVLWI is shown in Appendix 1. The current algorithm calculates intrathoracic blood volume (ITBV) from GEDV × 1.25. This assumption however, is debatable in both children and adults [16,23].

PiCCO measurements were performed by the attending critical care physician or experienced PICU nurses. A measurement was done by the subsequent injection of four boluses of ice-cold saline (3 to 5 ml, dependent on patient weight) through the central venous catheter. The PiCCO device was connected to a laptop PC for storage of data using the special PiCCOwin software (Pulsion, Munich, Germany). In this way all thermodilution curves and hemodynamic data were stored automatically for analysis afterwards. The software also stores the basic measurements that are needed for calculating EVLWI and GEDVI (mean transit time and down slope time) (Appendix 1). Erroneous measurements detected by clearly abnormal thermodilution curves including the cross-talk phenomenon were deleted afterwards [24]. A measurement was only accepted with a minimum of three reliable injections. EVLWI was calculated afterwards according to the calculations shown in Appendix 1 and indexed to actual body weight.

Cardiac output is expressed in liters per minute and indexed to body surface area (l/min/m^2^). Global end diastolic volume is expressed in milliliters and indexed to body surface area (ml/m^2^). Extravascular lung water is also expressed in milliliters and indexed to body weight (ml/kg).

### Chest x-ray

The chest x-rays were obtained in anteroposterior direction with the patient in supine position using a digital imaging system. The required energy (kV) was dependent on body weight and age and the actual x-ray was taken during maximal inspiration. The chest x-rays were analyzed on a dedicated digital radiology workstation with which, among others, brightness and contrast can be modified. Two radiologists with special pediatric expertise used the scoring system designed by Halperin *et al *(Table 1) [25]. This scoring technique divides the lungs into six regions. Right upper lobar, right perihilar, right lower, left upper, left perihilar and left lower lobar. The pulmonary regions are each scored using a semi-continuous scoring system consisting of 0 to 65 points. A score of 0 points indicates no signs of edema whereas a value of 65 represents severe edema. The points for the six regions are summed to construct the total score. In this way the total score ranges between 0 and 390 points. When a lung region could not be assessed because of atelectasis it was rated the mean value of the other two regions on the same side.

**Table 1:** Chest x-ray scoring system for quantification of pulmonary edema

Score (points)	Edema severity scoring
0	normal
10	mild pulmonary vascular congestion
20	moderate pulmonary vascular congestion
30	severe pulmonary vascular congestion
40	interstitial edema without septal lines
45	interstitial edema with septal lines
50	mixed interstitial and alveolar edema with some sparing of pulmonary region
55	mixed interstitial and alveolar edema involving entire region
60	alveolar edema with sparing of pulmonary region
65	alveolar edema involving entire pulmonary region

Based upon Halperin *et al *[25].

The radiologists were unaware of other patient characteristics but also unaware of the score of the other radiologist. Afterwards the inter-observer variability was calculated using concordance correlation and weighted kappa. The mean total scoring of the two radiologists was used to compare the chest x-ray score with the other recorded variables.

### Statistics

The correlation between EVLWI, chest x-ray score and surrogate markers of lung edema is unknown in children. We considered a correlation coefficient >0.6 as clinically relevant. With an alpha error of 0.05 and a power of 80%, a sample size of 19 would be necessary. This would require measurements from at least 19 individual patients. Because the correlation coefficient was essentially unknown we aimed for more than 20 children, including multiple measurements per patient.

All data were tested for normality using the d'Agostino Pearson test. The Pearson correlation coefficient was used for data with normal distribution and the Spearman correlation coefficient for data where normality was rejected. Variables are presented with median (interquartile range) except when specifically mentioned otherwise. Correlation and scatterplots were calculated and constructed using all separate measurements. For comparison of EVLWI and chest x-ray scores with patient characteristics, the mean values per patient were taken unless mentioned otherwise.

Data were stored in Excel software (Microsoft, Redmond WA, USA). Statistical calculations were performed using MedCalc 10 (MedCalc Software, Mariakerke, Belgium).

## Results

A total of 124 combined measurements from 27 patients were collected. After primary analysis four measurements were rejected because data were missing due to a storage failure, five measurements were excluded because the time interval between various parameters was more than three hours, twelve measurements were excluded because of an abnormal thermodilution curve. Consequently 103 measurements from 24 patients were eligible for final analysis of which 22 patients had serial measurements. Two patients died (8%). Twelve registrations started on Day 0, four on Day 1, five on Day 2 and three after Day 2.

The number of measurements per patient was 1 to 14 with a mean of 4.3 measurements per patient. Only five patients did not receive vasoactive support (24 measurements). All other patients were treated with dobutamine, milrinone or nor-epinephrine.

Individual patient characteristics are shown in Table 2. All children had normal body proportions. Table 3 shows the median values per variable. Of a total of 618 pulmonary regions (three per side in 103 patients) for the chest x-ray scoring method, the first radiologist could not score 20 regions (3.2%) and the second 17 regions (2.8%) because of atelectasis. The chest x-ray score ranged from 30 to 360 points with a median value of 133. The mean difference between left and right lung scoring was 1.7 (SD 4.8) for radiologist 1 and 6 (SD 9.5) for radiologist 2. The mean difference between the scoring of the two radiologists was 11.2 points with a range of -180 to +240 and an SD of 63.4. The concordance correlation between the two radiologists showed an r of 0.73 with 95% confidence interval of 0.63 to 0.81. The weighted kappa was 0.53 with standard error of 0.05.

**Table 2:** Patient characteristics per patient

Patient	Gender	Age	Weight	Diagnosis	Length of PICU stay	Ventilator days	Probability of death PRISM II	Probability of death PIM	Outcome
number	male/female	months	kg		days	days	%	%	
1	F	24	14	Near Drowning	19	17	85	60	survived
2	F	83	18	Reconstruction of pulmonary artery	18	16	7	6	survived
3	F	23	14	Abdominal surgery	5	3	78	17	survived
4	F	9	85	RSV	16	15	4	11	survived
5	F	31	16	Meningococcal disease	6	5	22	19	survived
6	F	2	48	Arterial switch operation	13	10	39	3	survived
7	F	5	71	Tetrology of Fallot repair	16	14	18	3	survived
8	F	8	65	Reconstruction of pulmonary artery	2	1	2	1	survived
9	M	4	44	VSD repair	13	5	26	1	survived
10	M	36	15	Meningococcal disease	5	4	8	7	survived
11	M	6	9	Meningococcal disease	5	4	9	8	survived
12	F	14	10	Meningococcal disease	4	3	28	9	survived
13	M	7	9	Inborn error of metabolism	12	5	88	3	survived
14	F	4	54	Post cardiac surgery	3	20	29	5	survived
15	M	17	12	Meningococcal disease	13	9	37	53	survived
16	M	24	13	Cardiac shock	20	9	31	28	survived
17	M	8	9	Pneumonia	20	15	5	4	survived
18	F	8	8,4	Status epilepticus	8	4	2	7	survived
19	F	28	10	Post CPR	4	3	70	46	survived
20	M	27	16	Meningococcal disease	6	5	61	63	survived
21	F	7	8	Shock/coma	12	7	54	24	survived
22	M	43	16	Septic shock	4	4	86	63	died
23	F	33	12	Septic shock	5	3	3	23	survived
24	M	32	152	Post CPR	18	16	40	2	died

**Table 3:** Values of several measurements

Variable	Value
MAP (mmHg)	65 (57 to 76)
Heart rate (bpm)	139 (118 to 153)
Cardiac index (l/min/m^2^)	4.00 (3.17 to 5.19)
GEDVI (ml/m^2^)	432 (369 to 528)
EVLWI (ml/kg)	16 (13 to 121)
Chest x-ray score	133 (90 to 204)
A-a gradient (mmHg)	119 (74 to 168)
PaO_2_/FiO_2_(mmHg)	283 (226 to 374)
PEEP (cmH_2_O)	6 (5 to 8)

median (interquartile range)

**Figure 1:**
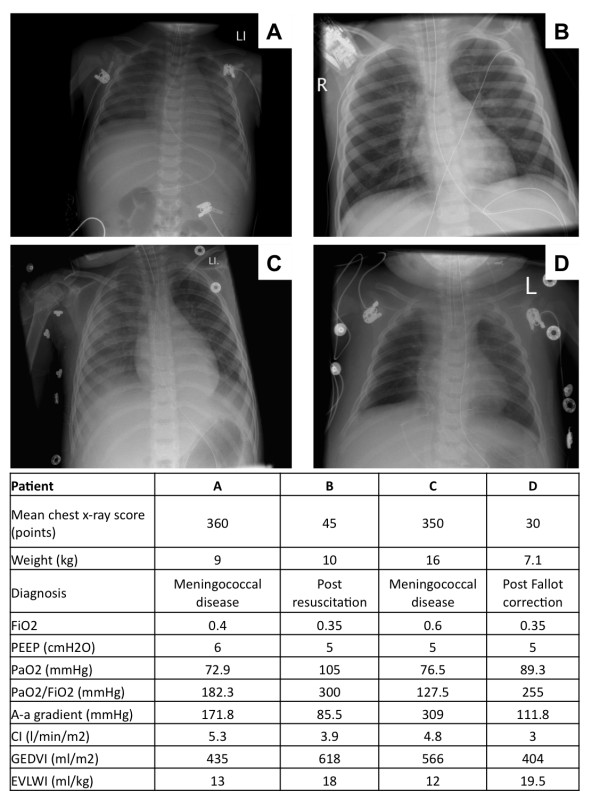
**Example of chest x-rays and related variables in four children with the lowest and highest chest x-ray score**.

**Figure 2:**
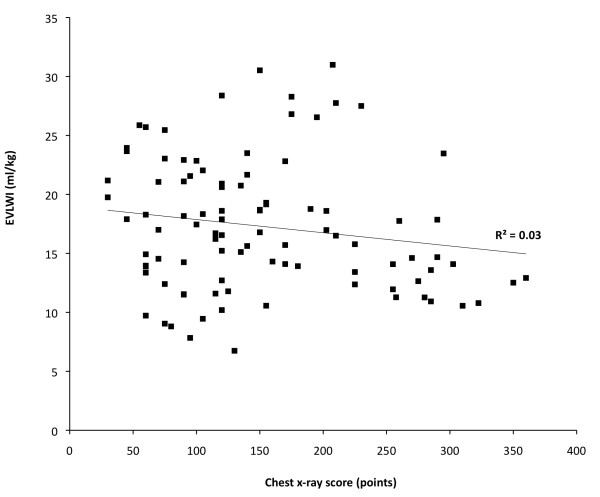
**Correlation between EVLWI and chest -x-ray score**.

Figure 1 shows four examples of the two lowest and the two highest chest x-ray scores and concomitant collected variables. Figure 2 shows the scatterplot of the chest x-ray score and EVLWI. There was no significant correlation between chest x-ray score and EVLWI. Also there was no significant correlation between EVLWI and the individual chest x-ray score by the radiologists.

Median PEEP level was 6 cmH_2_O (range 3 to 15). The PEEP level did not correlate with EVLWI, chest x-ray score, PaO_2_/FiO_2 _ratio or the A-a gradient.

There was no correlation between the mean chest x-ray score or EVLWI and severity of illness, length of stay, ventilator days, use of vasoactive medication, P/F ratio and A-a gradient (Table 4). Subsequently we determined these parameters per admission day. For the day of admission, the day of admission and the first day combined or the first three days, this did not change the results.

**Table 4:** Correlation between EVLWI, chest x-ray score and several relevant parameters

	EVLWI	Chest x-ray score
Age	-0.67 (<0.001)	-0.04 (0.724)
Body height	-0.80 (<0.001)	0.02 (0.4)
Ventilator days (days)	-0.009 (0.965)	-0.038 (0.86)
PIM score	-0.384 (0.064)	0.056 (0.795)
PRISM II score	-0.169 (0.429)	0.262 (0.216)
GEDVI	0.015 (0.946)	0.299 (0.156)
A-a gradient	-0.035 (0.866)	0.053 (0.799)
PaO_2_/FiO_2_	0.194 (0.364)	-0.087 (0.685)
EVLWI		-0.222 (0.296)

correlation coefficient with *P*-value

We also analyzed serial measurements per patient and found no correlation between changes in EVLWI on the one hand and changes in chest x-ray score, P/F ratio or A-a gradient on the other.

**Figure 3:**
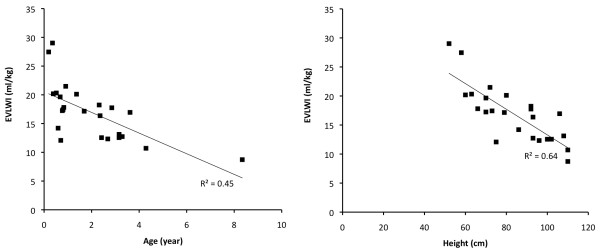
**Correlation between age and body height and EVLWI**.

The correlation between EVLWI and age is shown in Figure 3. The correlation coefficient between age and EVLWI was -0.67 (95% CI -0.85 to -0.36; *P *<0.001) and between EVLWI and height -0.80 (95% CI -0.91 to -0.59; *P *<0.0001). The chest x-ray score was not correlated with age or height.

## Discussion

This study shows that the measurement of extravascular lung water index does not correlate with a chest x-ray scoring system for quantification of pulmonary edema in critically ill children. Neither EVLWI nor the chest x-ray score correlated with markers of oxygenation.

The laboratory gold standard for the measurement of lung water is the postmortem gravimetric technique [26,27]. The clinical gold standard is the transpulmonary double indicator technique (TPDD) using injections of ice-cold indocyanine green (ICG) through a central venous catheter and an arterial catheter capable of detecting temperature and ICG concentration. Its accuracy has been demonstrated in animal studies [23,28]. However since the TPDD technology requires a rather large introducer sheath and several injections of ICG it has been replaced by the easier to apply TPTD technique. Validation of the TPTD technique has been performed in various animal experiments against the gravimetric technique. In general an acceptable accuracy was found although TPTD overestimates true EVLWI and is less reliable compared to TPDD [23,28-32]. In a recent study in adults a very close relation between EVLWI measured with TPTD with postmortem lung-weight (r^2 ^= 0.91) was demonstrated [33].

The calculation of EVLWI requires two variables: intrathoracic thermal volume (ITTV) and intrathoracic blood volume (ITBV) (Appendix 1). ITTV is directly measured using the TPTD technique and is not considered to be a factor for erroneous measurements. ITBV is directly measured using the TPDD technology but cannot be measured using the TPTD technique. Instead, GEDV is measured. Based upon a study by Sakka *et al *the relation between ITBV and GEDV in adults is reflected by the factor 1.25 [34]. The constant relationship between the two suggests that blood volume of the lung is linearly related to blood volume in the heart and great vessels. However it has been shown in adult patients that the relation between the two can vary [23].

Validation of EVLWI in children is more complicated. Clinically, the TPTD technique can only be compared to TPDD. We have previously shown in a small subset of patients that TPTD is generally reliable in children [16]. However, our study also showed that, like in adults, the relation between GEDV and ITBV is not always reflected by the factor of 1.25. We have shown that this factor is negatively correlated to body weight (r^2 ^= 0.52). Therefore it is possible that in children of variousages a different factor for the relation between ITBV and GEDV should be used. From a physiological viewpoint this looks attractive since, similar to the development of the lung, the relative blood volumes in the lung, heart and great vessels may change during growth. Also in this small group of relatively healthy patients the values of EVLWI were much higher than general adult values. Other studies have confirmed the higher values of EVLWI in younger children [17,18]. The present study shows that EVLWI values were much higher compared to adult values. Again we found a significant correlation between age (or height) and EVLWI. This shows that lung water index is an age dependent variable and that current adult normal values are not applicable to children. As only EVLWI was related to age this could explain the lack of correlation between EVLWI and the other variables.

The reason for the apparent higher values of EVLWI in younger children is not clear. Several explanations should be mentioned. First, EVLWI values could be falsely high but this is unlikely regarding our previous study [16]. Second, the total body water content is higher. Total body water decreases approximately by 15% during childhood [35]. Third, younger children may require a higher conversion factor when calculating ITBV from GEDV. Fourth, the relation between lung tissue mass and lung air volume is different in younger children (more tissue mass compared to air volume).

Contrary to the results in adults, there was no significant correlation between the PaO_2_/FiO_2 _ratio or A-a gradient and EVLWI [4,36]. Remarkably there was also no correlation between the chest x-ray score and the PaO_2_/FiO_2 _ratio or A-a gradient.

Several studies in adults also tried to correlate EVLWI, measured with the TPDD technique, with different types of chest x-ray score. In critically ill adults the results showed an r^2 ^between 0.2 and 0.7 or no correlation at all [13,25,37-39]. We found only one study that compared lung water in children with a chest x-ray score. In this small study using a different EVLW technique also no correlation between EVLWI and the chest x-ray was observed [40].

The radiographic determination of pulmonary edema may have several advantages over the dilution technique. It may detect edema in non-perfused regions while the dilution technique is dependent on an equal perfusion of all lung parts [41]. It is questionable whether the radiographic images reflect the same fluid collections that are measured with TPTD or TPDD. One may also argue that the fluid visible on the chest x-ray may not be measured with EVLWI because the indicator is unable to reach these collections (for example, alveolar or pleural fluids). No chest x-ray scoring system has been validated up till now. Finally, this study showed that even if chest x-rays are assessed by two experienced pediatric radiologists, the inter-observer agreement is still moderate. Finally it is also possible that changes in the chest x-ray appearance of pulmonary edema develop slowly compared to the EVLWI lung water measurement. Thereby the two estimates are not always synchronized.

Based upon this study it is questionable if a routine chest x-ray in critically ill children is justified to quantify the amount of pulmonary edema. This is in accordance with other studies considering the clinical value of routine chest x-rays in adults and children [42-46]. With regard to EVLWI measurements we believe, at present, that EVLWI in children should be studied further before it can be coupled to clinical decisions. Possible studies include the collection of normal values in relatively healthy children and pediatric animal studies validating EVLWI to gravimetry. The lack of age-related normal values makes comparing subgroups with normal or increased EVLWI difficult. Therefore it seems attractive to study other measurement methods for the determination of lung water. Ultrasound could be a reasonable alternative to chest x-ray for the determination of lung water although there are currently no available data in children [47-49].

Several limitations of our study should be noticed. We collected all relevant data within a relatively small time frame, but especially in small children, oxygenation may change rapidly. However, we assume that the EVLWI and the chest x-ray score do not vary significantly within a three-hour time frame since all data were collected under stable conditions. The chest x-ray score was not specifically designed for children but there is no reason why this is essentially different between adults and children. Also, the inter-rater agreement between the two radiologists was only moderate and individual scores were also not correlated with EVLWI. The reliability of EVLWI measurements decreases with pulmonary vascular obstruction including hypoxic pulmonary vasoconstriction and focal lung injury [41]. Also, high PEEP levels may obstruct small pulmonary vessels [50], although in our study the mean PEEP level was only 6.7 (SD 2.8) cmH_2_O. Pulmonary ventilation/perfusion mismatch may have been present in some children but there were no clinical signs of severe pulmonary perfusion abnormalities (like pulmonary emboli). The lack of correlation between EVLWI and chest x-ray score could also be explained by the diverse nature of the underlying pulmonary conditions. However, this study was deliberately performed in a general and mixed population of critically ill children to study the usefulness in every day practice. A more uniform patient group could have changed the results although in individuals there was also no correlation between measured variables over time. Not all measurements were started on the day of admission to the PICU. If possible, future studies should include measurements started on the same moment relative to the start of disease.

Another concern is the use of femoral venous catheters in some children. Because in these situations the route of the indicator is prolonged compared to catheters inserted in the upper body this may influence the mean transit time and thereby the measurement of EVLWI. However, we have shown earlier that EVLWI measurement was not different when comparing injection of the indictor in the right atrium compared to the femoral vein [16].

The fact that EVLWI in children is higher compared to adults and most importantly that this effect is age- or length-related makes this value difficult to interpret

## Conclusions

We conclude that extravascular lung water index measurements in a general population of critically ill children using the transpulmonary thermodilution technique do not correlate with a chest x-ray score of pulmonary edema. Neither lung water index nor the chest x-ray score of pulmonary edema correlates with markers of oxygenation, severity of illness or PICU length of stay.

## Key messages

• Extravascular lung water index measured in critically ill children using the transpulmonary thermodilution technique does not correlate with a chest x-ray score of pulmonary edema.

• Extravascular lung water in critically ill children does not correlate with parameters of oxygenation.

• A chest x-ray score of pulmonary edema in critically ill children does not correlate with parameters of oxygenation.

• In children extravascular lung water is inversely related to age (or body height).

• Further studies are needed before lung water can be used in pediatric clinical guidelines.

## Abbreviations

BSA: body surface area; CI: cardiac index; CO: cardiac output; CVP: central venous pressure; DAP: diastolic arterial pressure; EVLW: extravascular lung water; EVLWI: extravascular lung water index; FiO_2: _inspired oxygen concentration; GEDV: global end diastolic blood volume; GEDVI: global end diastolic blood volume index; HR: heart rate; ICG: indocyanine green; ITBV: intrathoracic blood volume; ITBVI: intrathoracic blood volume index; ITTV: intrathoracic thermal volume; kV: required energy; MAP: mean arterial pressure; PEEP: positive end expiratory pressure; RQ: respiratory quotient; SAP: systolic arterial pressure; TPDD: transpulmonary double indicator dilution technique; TPTD: transpulmonary thermodilution technique.

## Competing interests

The authors declare that they have no competing interests.

## Authors' contributions

JL designed the study, performed all statistics and wrote the manuscript. LD performed research in finding a suitable chest x-ray scoring system and subsequently scored the chest x-rays. She also collected the scoring from a colleague. AH assisted in the design of the study and assisted in collecting lung water data. JH assisted in the writing of the manuscript and supervised the research project.

## Appendix 1. Calculation of lung water index

### General

The required parameters for calculating lung water index are:

1. Cardiac output (CO) in l/min

2. Mean transit time (MTt) in sec

3. Mean downslope time (DSt) in sec

4. Body weight (kg)

### Calculations

- Intrathoracic thermal volume (ITTV) = CO × MTt × 1,000/60

- Pulmonary thermal volume (PTV) = CO × DSt × 1,000/60

- Global end diastolic volume (GEDV) = ITTV - PTV

- Intrathoracic blood volume (ITBV) = GEDV × 1.25

- EVLW = ITTV - ITBV

- EVLWI = EVLW/body weight
